# Syphilitic Joint Disease

**Published:** 1903-12-26

**Authors:** 


### SYPHILITIC JOINT DISEASE.

Mr. Percy Paton,[1] writing on this subject, states that though uncommon, the condition occurs somewhat more frequently than is generally supposed and is often overlooked or mistaken for some other affection. Of joint lesions in secondary syphilis he describes three varieties (1) arthralgia, (2) synovitis, and (3) hydrarthrosis. The first, in which pain increasing at night and without limitation of movement is almost the only symptom, occurs early, sometimes before the secondary eruption. Synovitis, a more common variety, may simulate rheumatism or come on without warning as an effusion with some local tenderness and pain which is not increased by movement. Hydrarthrosis in secondary syphilis nearly always affects the knee and occurs late in this stage; its onset is gradual and painless. Five varieties of tertiary syphilitic joint lesion are described. (1) Synovitis, either acute or chronic, in which the symptoms are those of a painless osteoarthritis. (2) Gummatous synovitis, in which condition there may be either much or little fluid; the synovial membrane is puckered and lumpy, pain is chiefly at night and not starting in character; relapses are frequent. (3) In some cases bones or cartilages are primarily diseased. Though there is little pain there is much limitation of movement, and thickening of bone ends with lipping may be a marked feature. Ankylosis is to be feared if this condition is not recognised and treated early. (4) A gumma near a joint may perforate the joint and cause secondary effusion. A much more serious complication sometimes occurs when a gumma bursts externally and also perforates the joint causing septic arthritis followed by destruction of the joint. (5) Ankylosis may occur in any case of gummatous joint affection which is not recognised and treated early, and especially when the joint is kept rigidly at rest.

In hereditary syphilis the following six varieties of joint disease have been described:—(1) Simple synovial effusion, which is fairly common, affects the knee, and is often associated with interstitial keratitis. (2) Joint complication of syphilitic epiphysitis is characterised by much boggy swelling round the joint and loss of function in the limb. (3) Primary gummatous affection of the synovial membrane; and (5) osteitis and gummatous synovitis which have the same symptoms in the hereditary as in the acquired disease. (4) Osteitis, in which the ends of the bones are enlarged and fluid is poured out into the synovial cavity, is a painless condition, amenable to treatment, frequently relapsing. (6) Deforming arthritis is rarely seen. Osteophytic outgrowths limit the joint movement and sometimes similate dislocation rather than disease.

The special diagnostic points in all syphilitic arthropathies, apart from other evidence of syphilis, are the slight pain on movement, the mild constitutional disturbance, the fact that the knee is the joint most often affected, and the satisfactory results of anti-syphilitic treatment.

Treatment consists in the exhibition of mercury and iodides, counter-irritation, passive and active movement, and massage.

[1] British Medical Journal, Nov. 28, 1903.

